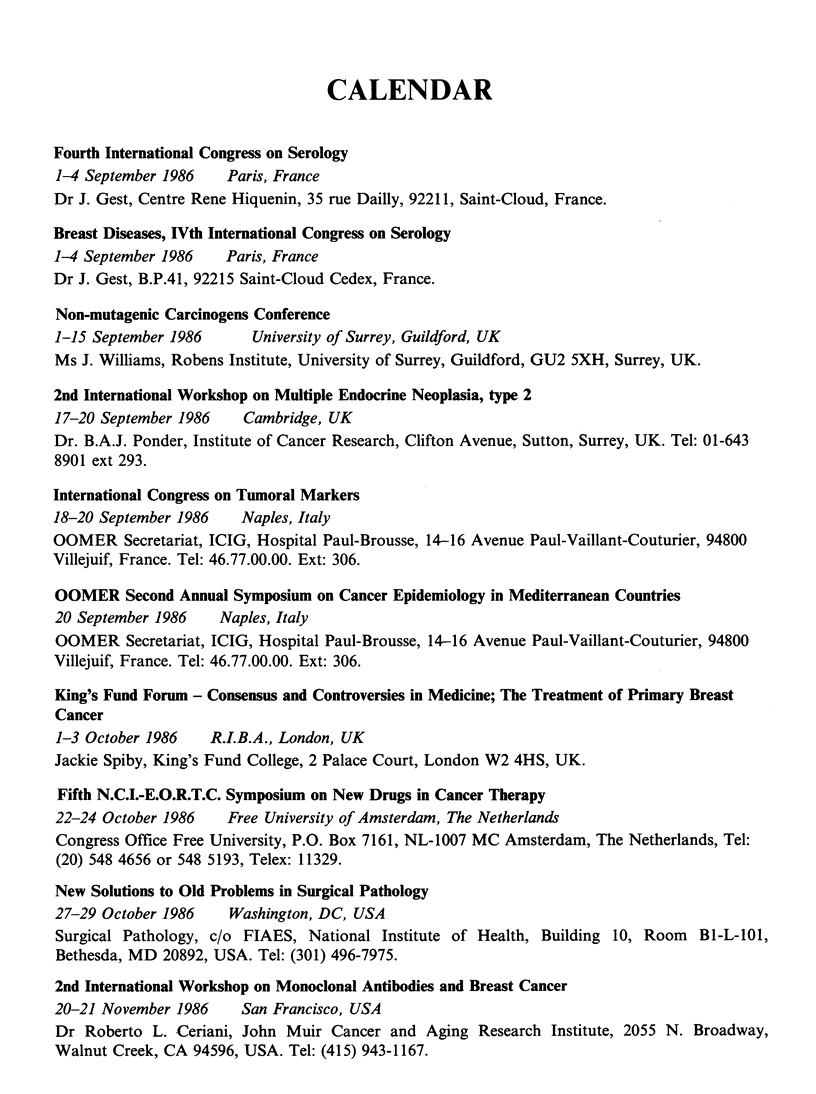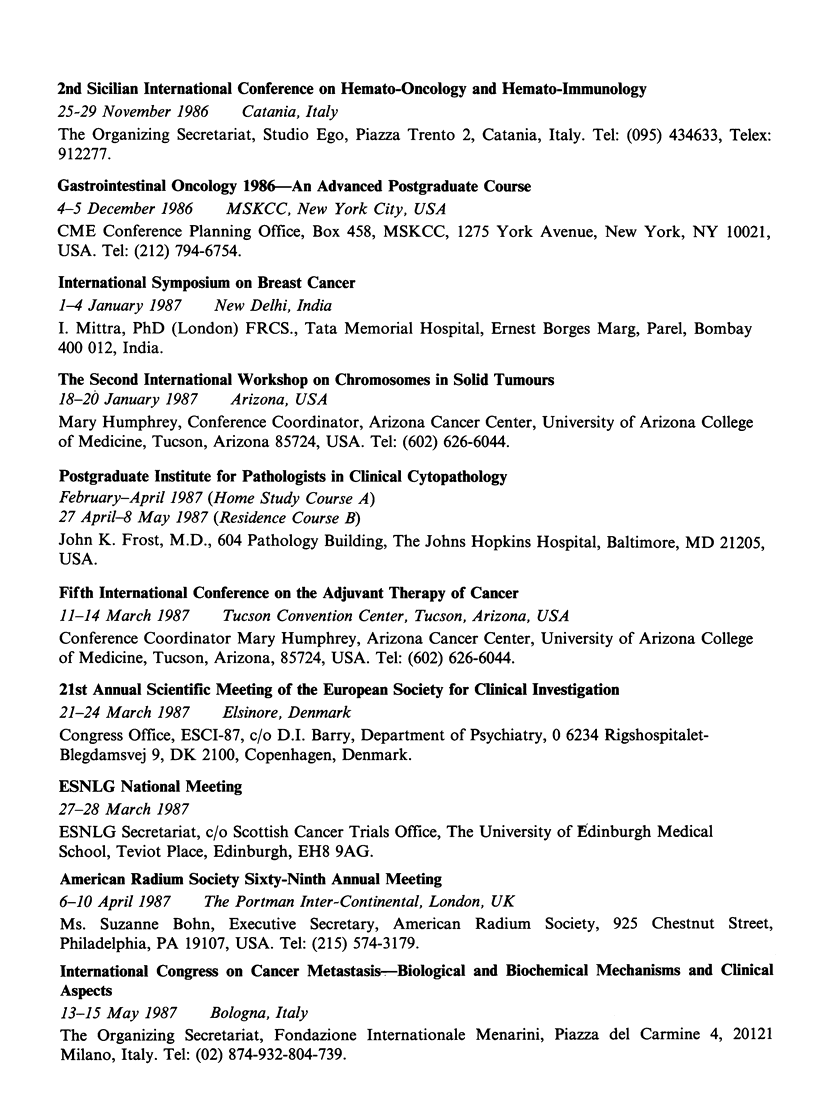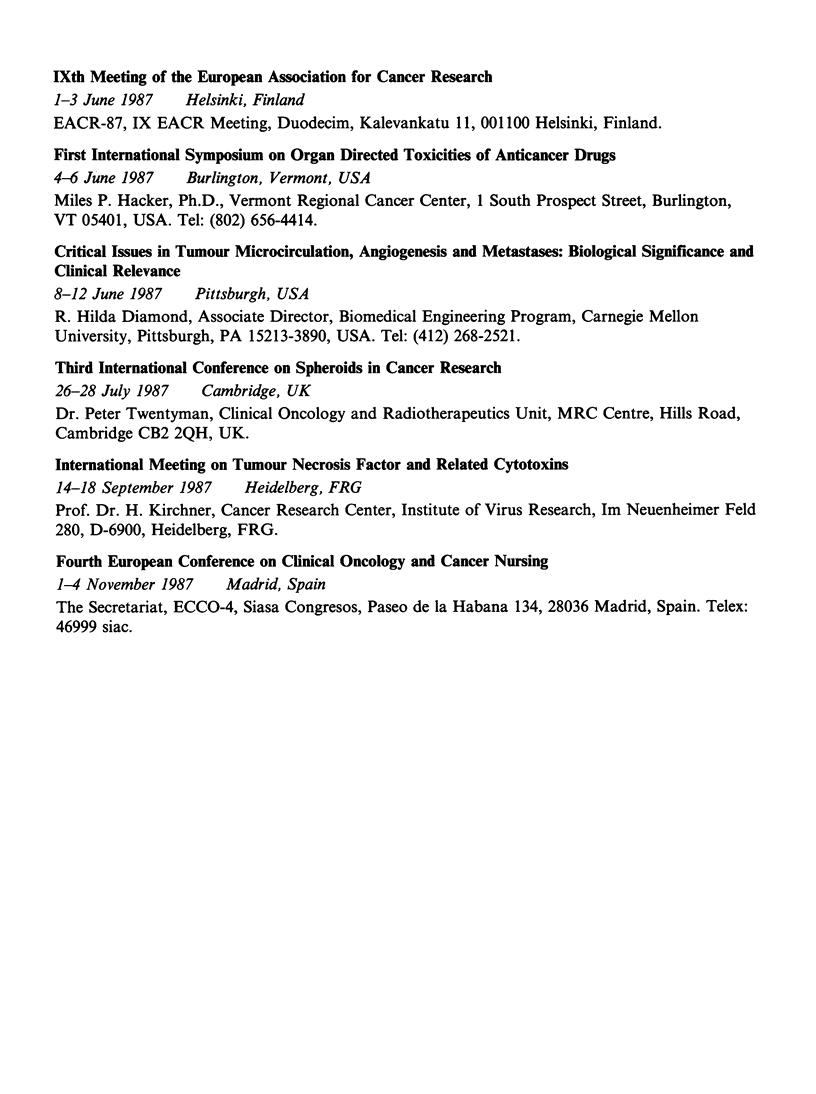# Calendar

**Published:** 1986-09

**Authors:** 


### CALENDAR

**Fourth International Congress on Serology**

*1–4 September 1986 Paris, France*

Dr J. Gest, Centre Rene Hiquenin, 35 rue Dailly, 92211, Saint-Cloud, France.

**Breast Diseases, IVth International Congress on Serology**

*1–4 September 1986 Paris, France*

Dr J. Gest, B.P.41, 92215 Saint-Cloud Cedex, France.

**Non-mutagenic Carcinogens Conference**

*1–15 September 1986 University of Surrey, Guildford, UK*

Ms J. Williams, Robens Institute, University of Surrey, Guildford, GU2 5XH, Surrey, UK.

**2nd International Workshop on Multiple Endocrine Neoplasia, type 2**

*17–20 September 1986 Cambridge, UK*

Dr. B.A.J. Ponder, Institute of Cancer Research, Clifton Avenue, Sutton, Surrey, UK. Tel: 01-643 8901 ext 293.

**International Congress on Tumoral Markers**

*18–20 September 1986 Naples, Italy*

OOMER Secretariat, ICIG, Hospital Paul-Brousse, 14–16 Avenue Paul-Vaillant-Couturier, 94800 Villejuif, France. Tel: 46.77.00.00. Ext: 306.

**OOMER Second Annual Symposium on Cancer Epidemiology in Mediterranean Countries**

*20 September 1986 Naples, Italy*

OOMER Secretariat, ICIG, Hospital Paul-Brousse, 14–16 Avenue Paul-Vaillant-Couturier, 94800 Villejuif, France. Tel: 46.77.00.00. Ext: 306.

**King's Fund Forum – Consensus and Controversies in Medicine; The Treatment of Primary Breast Cancer**

*1–3 October 1986 R.I.B.A., London, UK*

Jackie Spiby, King's Fund College, 2 Palace Court, London W2 4HS, UK.

**Fifth N.C.I.-E.O.R.T.C. Symposium on New Drugs in Cancer Therapy**

*22–24 October 1986 Free University of Amsterdam, The Netherlands*

Congress Office Free University, P.O. Box 7161, NL-1007 MC Amsterdam, The Netherlands, Tel: (20) 548 4656 or 548 5193, Telex: 11329.

**New Solutions to Old Problems in Surgical Pathology**

*27–29 October 1986 Washington, DC, USA*

Surgical Pathology, c/o FIAES, National Institute of Health, Building 10, Room B1-L-101, Bethesda, MD 20892, USA. Tel: (301) 496-7975.

**2nd International Workshop on Monoclonal Antibodies and Breast Cancer**

*20–21 November 1986 San Francisco, USA*

Dr Roberto L. Ceriani, John Muir Cancer and Aging Research Institute, 2055 N. Broadway, Walnut Creek, CA 94596, USA. Tel: (415) 943-1167.

**2nd Sicilian International Conference on Hemato-Oncology and Hemato-Immunology**

*25-29 November 1986*     *Catania, Italy*

The Organizing Secretariat, Studio Ego, Piazza Trento 2, Catania, Italy. Tel: (095) 434633, Telex: 912277.

**Gastrointestinal Oncology 1986—An Advanced Postgraduate Course**

*4–5 December 1986*     *MSKCC, New York City, USA*

CME Conference Planning Office, Box 458, MSKCC, 1275 York Avenue, New York, NY 10021, USA. Tel: (212) 794-6754.

**International Symposium on Breast Cancer**

*1–4 January 1987*     *New Delhi, India*

I. Mittra, PhD (London) FRCS., Tata Memorial Hospital, Ernest Borges Marg, Parel, Bombay 400 012, India.

**The Second International Workshop on Chromosomes in Solid Tumours**

*18–20 January 1987*     *Arizona, USA*

Mary Humphrey, Conference Coordinator, Arizona Cancer Center, University of Arizona College of Medicine, Tucson, Arizona 85724, USA. Tel: (602) 626-6044.

**Postgraduate Institute for Pathologists in Clinical Cytopathology**

*February–April 1987* (*Home Study Course A*)
*27 April–8 May 1987* (*Residence Course B*)

John K. Frost, M.D., 604 Pathology Building, The Johns Hopkins Hospital, Baltimore, MD 21205, USA.

**Fifth International Conference on the Adjuvant Therapy of Cancer**

*11–14 March 1987*     *Tucson Convention Center, Tucson, Arizona, USA*

Conference Coordinator Mary Humphrey, Arizona Cancer Center, University of Arizona College of Medicine, Tucson, Arizona, 85724, USA. Tel: (602) 626-6044.

**21st Annual Scientific Meeting of the European Society for Clinical Investigation**

*21–24 March 1987*     *Elsinore, Denmark*

Congress Office, ESCI-87, c/o D.I. Barry, Department of Psychiatry, 0 6234 Rigshospitalet-Blegdamsvej 9, DK 2100, Copenhagen, Denmark.

**ESNLG National Meeting**

*27–28 March 1987*

ESNLG Secretariat, c/o Scottish Cancer Trials Office, The University of Edinburgh Medical School, Teviot Place, Edinburgh, EH8 9AG.

**American Radium Society Sixty-Ninth Annual Meeting**

*6–10 April 1987*     *The Portman Inter-Continental, London, UK*

Ms. Suzanne Bohn, Executive Secretary, American Radium Society, 925 Chestnut Street, Philadelphia, PA 19107, USA. Tel: (215) 574-3179.

**International Congress on Cancer Metastasis—Biological and Biochemical Mechanisms and Clinical Aspects**

*13–15 May 1987*     *Bologna, Italy*

The Organizing Secretariat, Fondazione Internationale Menarini, Piazza del Carmine 4, 20121 Milano, Italy. Tel: (02) 874-932-804-739.

**IXth Meeting of the European Association for Cancer Research**

*1–3 June 1987 Helsinki, Finland*

EACR-87, IX EACR Meeting, Duodecim, Kalevankatu 11, 001100 Helsinki, Finland.

**First International Symposium on Organ Directed Toxicities of Anticancer Drugs**

*4–6 June 1987 Burlington, Vermont, USA*

Miles P. Hacker, Ph.D., Vermont Regional Cancer Center, 1 South Prospect Street, Burlington, VT 05401, USA. Tel: (802) 656-4414.

**Critical Issues in Tumour Microcirculation, Angiogenesis and Metastases: Biological Significance and Clinical Relevance**

*8–12 June 1987 Pittsburgh, USA*

R. Hilda Diamond, Associate Director, Biomedical Engineering Program, Carnegie Mellon University, Pittsburgh, PA 15213-3890, USA. Tel: (412) 268-2521.

**Third International Conference on Spheroids in Cancer Research**

*26–28 July 1987 Cambridge, UK*

Dr. Peter Twentyman, Clinical Oncology and Radiotherapeutics Unit, MRC Centre, Hills Road, Cambridge CB2 2QH, UK.

**International Meeting on Tumour Necrosis Factor and Related Cytotoxins**

*14–18 September 1987 Heidelberg, FRG*

Prof. Dr. H. Kirchner, Cancer Research Center, Institute of Virus Research, Im Neuenheimer Feld 280, D-6900, Heidelberg, FRG.

**Fourth European Conference on Clinical Oncology and Cancer Nursing**

*1–4 November 1987 Madrid, Spain*

The Secretariat, ECCO-4, Siasa Congresos, Paseo de la Habana 134, 28036 Madrid, Spain. Telex: 46999 siac.